# Assessing the Hibernation Ecology of the Endangered Amphibian, *Pelophylax chosenicus* Using PIT Tagging Method

**DOI:** 10.3390/ani15243638

**Published:** 2025-12-17

**Authors:** Kwanik Kwon, Changdeuk Park, Jeongwoo Yoo, Nakyung Yoo, Keun-Sik Kim, Juduk Yoon

**Affiliations:** 1 Research Center for Endangered Species, National Institute of Ecology, Yeongyang 36531, Republic of Korea; econlearn@nie.re.kr (K.K.); chummaum@nie.re.kr (C.P.); wildlife3028@nie.re.kr (J.Y.); nkyoo96@nie.re.kr (N.Y.); kskim@nie.re.kr (K.-S.K.); 2 Department of Life Sciences, College of Natural Science, Yeungnam University, Gyeongsan 38541, Republic of Korea

**Keywords:** gold-spotted pond frog, hibernation, endangered amphibian, movement, ecology, PIT tagging

## Abstract

The Gold-spotted pond frog (*Pelophylax chosenicus* (Okada, 1931)), classified as an endangered species in South Korea, has experienced significant population declines due to habitat loss, primarily driven by agricultural expansion and urbanization. This study aimed to assess the hibernation ecology of *P. chosenicus* using PIT tagging technology, focusing on its hibernation behavior, environmental conditions, and implications for conservation and restoration. Over a three-year period (2021–2024), PIT tags were implanted in 408 frogs, enabling continuous monitoring of hibernation sites and individual characteristics. The results revealed that hibernation depths ranged from 1 to 23 cm, with deeper burrowing observed during colder months, indicating a temperature-dependent survival strategy. The soil temperature at hibernation sites remained consistently higher than ambient air temperature, suggesting an adaptive mechanism to avoid freezing conditions. Additionally, frogs exhibited a strong preference for hibernation sites near water bodies, where soil moisture levels were high, highlighting the importance of preserving suitable microhabitats for successful overwintering. These findings provide valuable insights into the hibernation ecology of *P. chosenicus* and are critical for guiding effective habitat restoration initiatives. Future research should investigate physiological adaptations to varying hibernation environments and the potential impacts of climate change may impact the survival of this endangered species.

## Simple Summary

This study investigated the hibernation ecology of the endangered Gold-spotted pond frog (*Pelophylax chosenicus*) using PIT tag tracking to support conservation efforts. By monitoring 49 hibernating individuals over a three-year period, we found the frogs burrowed at depths ranging from 1 to 23 cm. The frogs employed a behavioral strategy of digging deeper during colder periods, utilizing soil as a thermal buffer to maintain body temperatures above ambient air temperature. Furthermore, to prevent dehydration, they exhibited a strong preference for hibernation sites near the waterfront with high soil moisture content. We conclude that preserving microhabitats with adequate burial depth and high soil moisture near the waterfront is critical for the successful overwintering and subsequent conservation of *P. chosenicus*.

## 1. Introduction

Amphibians in temperate regions survive low winter temperatures either by developing freeze tolerance or avoid sub-zero temperatures [1,2]. In anurans, hibernation occurs in both aquatic and terrestrial environments. Terrestrial hibernation can be broadly divided into two strategies: individuals either burrow deeply into the soil and remain in unfrozen layers [3,4] or they remain at shallow depths while maintaining a freeze-tolerant state [2,5]. Most species of frogs hibernate underwater, some species burrow into the soil and hibernate on land, but only a few species demonstrate frost resistance [1,2]. Because hibernating frogs are rarely encountered without targeted survey methods, and because specialized techniques are required to locate them, knowledge of their hibernation ecology remains limited.

The Gold-spotted pond frog *Pelophylax chosenicus* is a small ranid species endemic to the western part of the Korean Peninsula. It primarily inhabits lowland agricultural landscapes, wetlands and reservoirs [6]. As this species relies heavily on rice paddies as its core breeding habitat, it has been severely impacted by agricultural chemicals and, more significantly, by habitat loss associated with urbanisation and land development [7]. Consequently, populations have declined markedly, leading to its current classification as class II endangered Wildlife in Korea. The species is also listed as Vulnerable on both the IUCN Red List and the Korean Red List. In response, conservation initiatives have been implemented in Korea, including the development of captive-breeding protocols and efforts to preserve genetic diversity.

Passive integrated transponder (PIT) tags are electronic devices that receive electrical signals from an external antenna via electromagnetic coupling and subsequently transmit a unique identification code, which can be detected by the same antenna [8]. Although their detection range is limited to approximately 1 m, this constraint enables accurate individual identification and precise localization. As a result, PIT tags have been widely employed in studies of survival, movement patterns, and population dynamics [9,10,11,12]. In amphibian research, PIT tags are well suited for long-term individual marking and have been successfully applied in studies worldwide [13,14]. In Korea, PIT tagging has also been utilized in ecological studies of reptiles [15].

Restoration of *P. chosenicus* requires an integrated approach that combines multiple research disciplines, in which ecological traits must be considered alongside captive-breeding efforts. Among these traits, hibernation plays a critical role in reproduction but has been largely overlooked in previous studies of this species. *P. chosenicus* is a semi-aquatic amphibian presumed to overwinter in both aquatic and terrestrial habitats; however, its hibernation ecology remains undocumented. While aquatic hibernation can be partially inferred through direct observation, terrestrial hibernation is difficult to confirm due to the species’ burrowing behavior into the soil. Although the precise mechanism of burrowing by *P. chosenicus* has not been confirmed in the field, evidence suggests that it likely employs its hind limbs. This inference is based on the general observation that more than 95% of burrowing anurans utilize a hindfeet-first digging pattern, which is unique among terrestrial vertebrates [16]. Furthermore, the use of hind limbs for burrowing was consistently observed during laboratory hibernation experiments involving this species.

In this study, we employed PIT tags to detect terrestrial hibernation in *P. chosenicus* and to characterise hibernacula and their associated environmental conditions. Given that PIT tags enable individual identification, we were able to track, body size and other phenotypic traits of each frog throughout the hibernation period, thereby facilitating longitudinal analyses at the individual level. Our objectives were threefold: to elucidate the hibernation ecology of *P. chosenicus*, to provide baseline data for the development of captive-breeding protocols, and to generate essential information for designing suitable microhabitats in the context of conservation planning and habitat creation or management for this endangered species.

## 2. Materials and Methods

### 2.1. Study Site and Period

**Figure 1:**
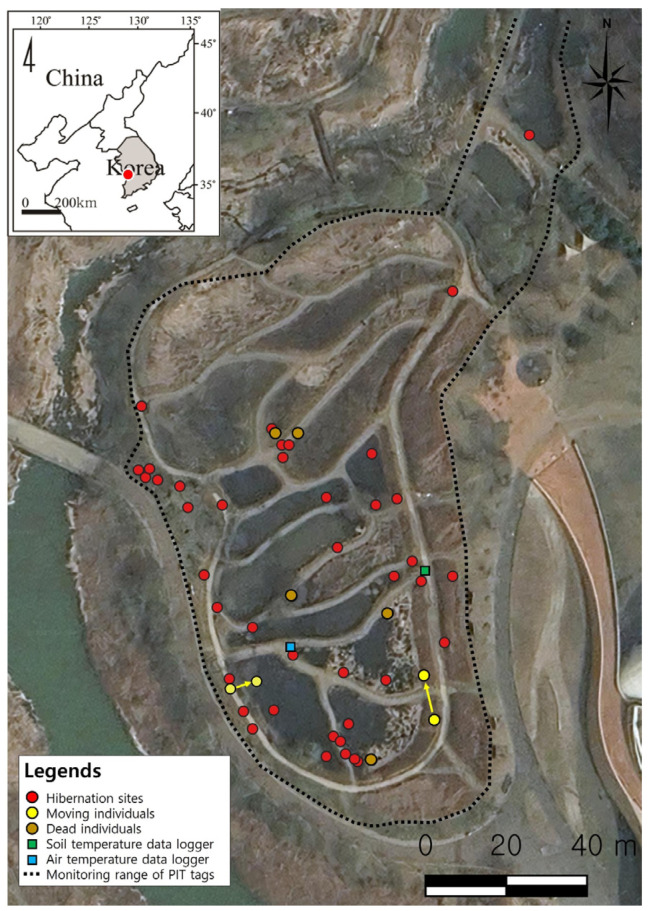
The map of location of *P. chosenicus* hibernation sites (Yellow dots indicate the locations of individuals that moved during hibernation). The yellow arrows indicate individuals that exhibited movement during the hibernation period.

The study was conducted at and around the Aquatic Botanical Garden (36°02′18.16″ N, 126°43′07.04″ E) located within the National Institute of Ecology (NIE), Seocheon, Republic of Korea (Figure 1). The area originally supported a natural population of *P. chosenicus*; however, construction activities at the NIE between July 2009 and December 2012 resulted in the translocations of frogs, after which records of the species became rare. In 2019, individuals produced through captive breeding at the NIE Research Center for Endangered Species were released to reestablish the local population. The Aquatic Botanical Garden consists of 16 artificial wetlands designed to resemble the Korean Peninsula, with a total area of 14,500 m^2^.

Hibernation surveys were conducted over three consecutive hibernation seasons from 2021 to 2024: November 2021–March 2022, November 2022–March 2023, and November 2023–March 2024. Survey periods were determined based on the known timing and thermal conditions associated with the onset and termination of hibernation in anurans incorporating buffer periods before and after the core hibernation season. All procedures were carried out in accordance with guidelines of the Institutional Animal Care and Use Committee of the National Institute of Ecology (Approval No. NIEIACUC-2021-024).

### 2.2. PIT Tagging and Monitoring

#### 2.2.1. PIT Tag Implantation

The hibernation study on *P. chosenicus*, a Class II Endangered Wildlife species in Korea, was conducted under permits issued by the Geum River Basin Environmental Office, Daejeon, Republic of Korea (Permit Nos. 2021-6 and 2022-58). We employed PIT telemetry, which enables individual identification of frogs. The PIT tags used (HDX, Oregon RFID, Portland, OR, USA) measured 12 mm in length, 2.12 mm in diameter, and weighed 0.1 g. These tags can be used semi-permanently because they receive an electrical signal from a receiver and verify the information. These tags are widely used in studies on small animals and have a detection range of approximately 30 cm with a handheld reader.

Frogs were captured manually during nocturnal surveys in the Aquatic Botanical Garden. For each individual, snout–vent length (SVL, mm) was measured to the nearest 0.01 mm using a digital vernier caliper (Mitutoyo, CD-10AX, Kawasaki, Kanagawa, Japan), and body weight (BW, g) was recorded to the nearest 0.1 g using an electronic balance (Qixindaojian Co. Ltd., Qingdao, China). PIT tags were implanted subcutaneously between the epidermis and dermis on the dorsal side of individuals with SVL ≥ 30 mm using a dedicated tag insertion needle. Following implantation, wounds were disinfected with povidone–iodine and sealed with a veterinary tissue adhesive (Vetbond Tissue Adhesive, 3M, St. Paul, MN, USA).

Tagged frogs were temporarily housed in well-ventilated 60 L PVC tanks (60 cm × 43 cm × 36 cm), with 10–20 individuals per tank, for approximately 24 h to monitor for tag loss or abnormal behaviour. Individuals showing no adverse effects were subsequently released at their original capture locations. PIT tagging was performed between April and October, prior to the onset of overwintering.

#### 2.2.2. Monitoring

Monitoring of *P. chosenicus* was conducted monthly. Each monitoring session consisted of three standardized steps: (1) locating individuals using a PIT antenna and receiver, (2) confirmed the hibernating individuals and recorded their body measurements to examine the physical changes that occurred during hibernation, and (3) characterising hibernation sites. Hibernacula were surveyed once per month throughout each hibernation season.

To locate hibernating frogs, we used an HPR Plus PIT tag reader and BP Plus portable antenna (Biomark, Boise, ID, USA). The terrestrial area of the Aquatic Botanical Garden was systematically scanned manually, covering the entire zone within approximately 20 m of the water’s edge. Coordinates of detected PIT tag signals were automatically recorded by the receiver, and each location was marked with a garden pick (plant label) for subsequent excavation.

At each signal detection point, soil was carefully excavated using a shovel and hand trowel. Once a frog was found, snout–vent length (SVL) and body weight (BW) were measured and the individual was returned to its original hibernaculum. At each hibernation site, burial depth (soil depth), soil temperature (Acuba CS-101, Shenzhen, China), soil moisture and soil pH (Takemura DM-5, Tokyo, Japan) were measured. After all measurements were completed, the excavated soil was replaced and the site was restored as closely as possible to its original condition.

To continuously monitor soil and air temperatures at the study site, HOBO data loggers (Onset, Bourne, MA, USA) were installed. For soil temperature, a pendant-type HOBO logger (UA-002-64) was buried at a depth of 30 cm at a hibernation site confirmed in 2021. For air temperature, a mounted HOBO logger (U23-001A) was installed in a well-ventilated location at a height of 50 cm above the ground near the centre of the study area. Temperature monitoring was conducted from November 2023 to March 2024, and data were downloaded using a coupler during each field visit.

### 2.3. Hibernation Temperature Experiments

To determine the temperatures thresholds at which *P. chosenicus* enters and terminates hibernation, we conducted both field observations and laboratory experiments. Field observations were carried out from November 2022 to March 2023. Within the known hibernation period, the date on which frogs were detected was considered the end of hibernation, and the first detection date in the following year was defined as the onset of emergence from hibernation. Temperatures on these dates were obtained from HOBO logger records for the corresponding days.

In the laboratory, hibernation behavior was monitored under controlled environmental conditions. Two plastic boxes (30 × 24 × 28 cm, 20 L) each filled with 20 cm of soil and covered with bark to minimize evaporation were placed in a programmable refrigerator (GMSR-322, GMS, Seoul, Republic of Korea). Females and males were housed separately in different boxes. The experiment lasted 50 days from 3 February to 25 March 2022. Experimental animals consisted of 35 adult frogs (20 females, 15 males) that had been captured in Asan City between 2018 and 2021 and introduced into the captive-breeding programme.

At the start of the experiment, the temperature was set at 18 °C and then decreased by 5 °C every 7 days. We recorded the temperature at which frogs began burrowing into the soil as the onset of hibernation. After maintaining the minimum temperature of 3 °C for 7 days, the temperature was increased again by 5 °C at 7-day intervals. When the temperature reached 18 °C, frogs were acclimated to room temperature for an additional 4 days, and Body measurements were taken before the experiment was terminated to examine physical changes during hibernation. Throughout the experiment, the photoperiod was maintained at 9L:15D.

### 2.4. Data Analysis

To characterise hibernacula of *P. chosenicus*, we examined relationships among seven variables: two biological variables (SVL and BW) and five environmental variables (soil moisture, soil depth, soil temperature, soil pH and average air temperature). Pearson’s correlation analysis was performed to assess linear relationships and overall interdependence among these variables. This analysis enabled evaluation of the associations between body size (SVL, BW) and hibernaculum conditions, as well as quantification of statistical correlations among environmental factors. Relationships were interpreted based on the magnitude and direction of correlation coefficients and their statistical significance (*p*-values).

To test whether hibernacula function as thermal buffers that protect frogs from external environmental fluctuations, we conducted paired *t*-tests and compared soil temperature within hibernacula with corresponding average air temperature, which represents conditions experienced by frogs above ground. All statistical analyses were performed in R (R version 4.4.2, R Project).

## 3. Results

### 3.1. Characteristics of Hibernation Sites

Over the three hibernation seasons, PIT tags were implanted in 77 frogs during the first year, 193 during the second year and 138 during the third year, resulting in a total of 408 tagged *P. chosenicus*. Among these, 49 individuals (12.0%) were successfully detected at hibernation sites through monitoring. By season, three individuals were detected in the first hibernation season, 31 in the second and 16 in the third; however, since one individual tagged in the second season was also recaptured in the third, the number of unique individuals in the third season was 15 (Table 1). Hibernation sites were located in terrestrial areas adjacent to the main habitat, the Aquatic Botanical Garden, where frogs had burrowed into the soil (Figure 1).

**Figure 2:**
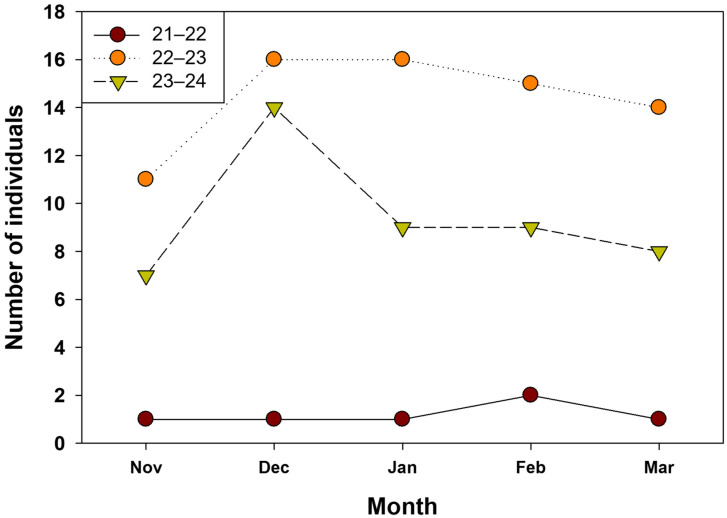
Number of hibernation sites detected by three study periods.

Among hibernating individuals, SVL ranged from 29.38 to 64.9 mm, with a mean of 45.5 ± 7.9 mm. BW ranged from 3.5 to 34.5 g, with a mean of 14.3 ± 8.7 g. Excluding the first year, when only three individuals were detected, mean monthly SVL in the second and third seasons ranged from 42.4 to 49.2 mm, and mean monthly BW ranged from 10.1 to 18.1 g, with no clear monthly patterns. The number of confirmed hibernation sites varied slightly among months; excluding data from the first year, hibernacula were first detected in November, peaked in December, and declined thereafter, with numbers decreasing around March (Figure 2).

**Figure 3:**
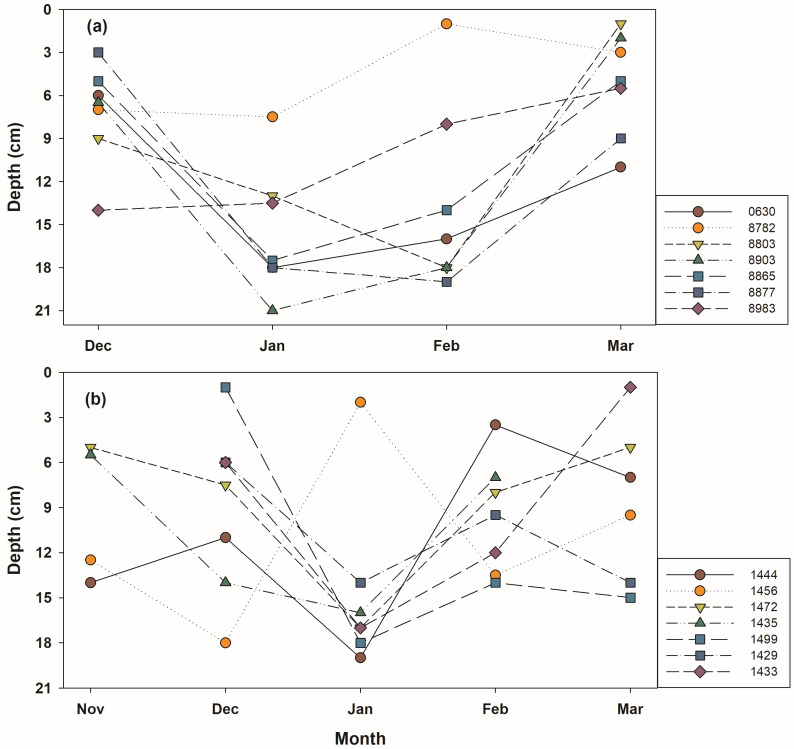
Monthly changes in hibernation depth are presented for individuals with more than four detections. The numbers represent the tag IDs of each individual ((**a**): 2022–2023, (**b**): 2023–2024).

**Figure 4:**
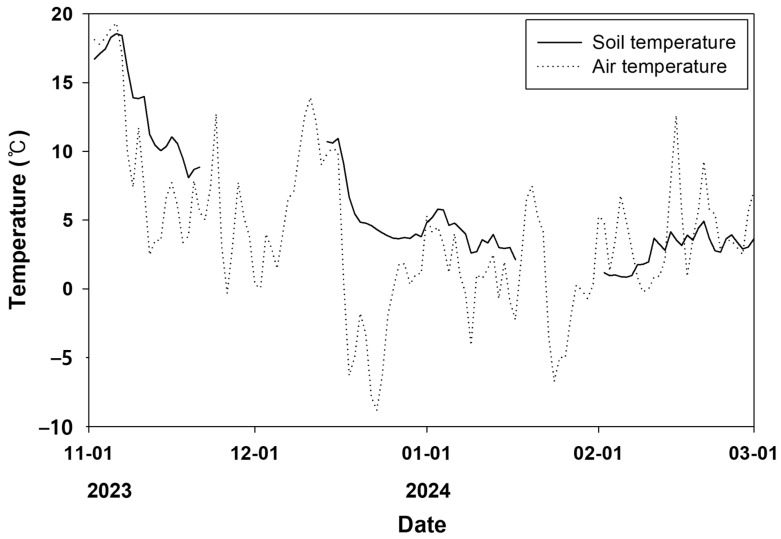
Air temperature and soil temperature changes in the hibernating area. Solid and dotted lines indicated changes of soil and air temperature, respectively.

**Figure 5:**
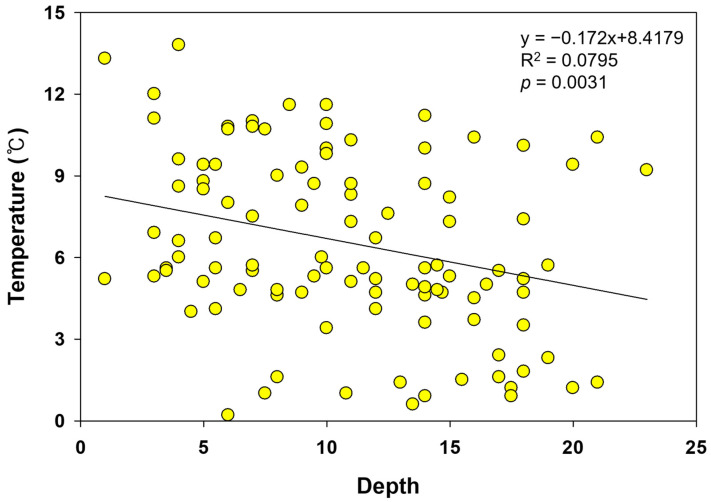
Scatterplot showing the correlation between soil temperature and hibernation depth. The solid line represents the linear regression.

Burial depth (soil above the dorsal part frog’s) at hibernation sites ranged from 1 to 23 cm, with a mean depth of 11.0 ± 5.3 cm (Table 1). Hibernation depth was not uniform and varied among individuals; however, frogs tended to burrow deepest in January, following the onset of hibernation in November, and then gradually move closer to the surface beginning in February (Table 1, Figure 3). Soil temperature at hibernation sites exhibited a pattern similar to that of air temperature (Figure 4). As air temperature decreased, soil temperature also decreased, and as air temperature increased, soil temperature increased. Until late January, when air temperatures remained low, soil temperature was consistently higher than air temperature; during the subsequent warming period, soil temperature tended to be lower than air temperature. The mean temperature at hibernacula (6.5 ± 3.3 °C) was significantly higher than the mean air temperature (4.5 ± 3.3 °C; paired *t*-test, *t* = 10.44, *p*-value < 0.01). Soil and air temperatures at hibernation sites were strongly positively correlated (Pearson *r* = 0.8126, *p*-value < 0.01; Table 2). A tendency for soil temperature to increase with increasing burial depth was also observed (Figure 5), suggesting that frogs burrow deeper in response to declining temperatures.

**Figure 6:**
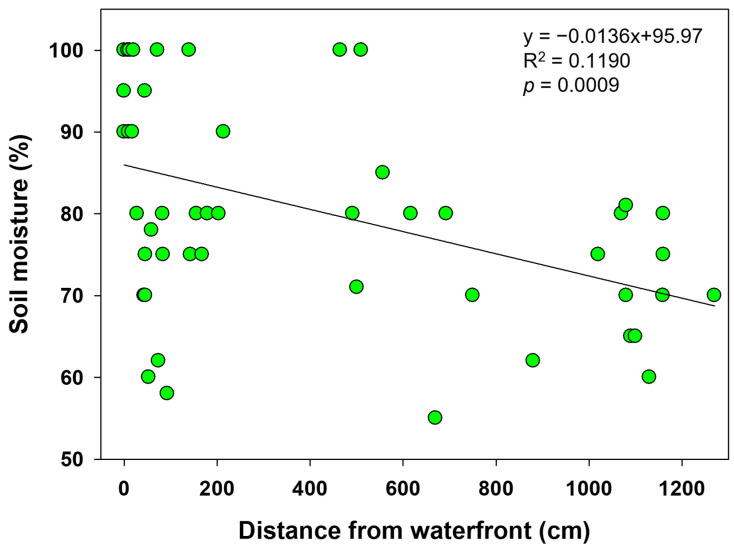
Scatterplot showing the correlation between the distance from waterfront and the soil moisture. The solid line represents the linear regression.

Mean soil pH at hibernacula was 6.3 ± 0.3. Soil moisture at hibernation sites averaged 78.1 ± 14.6%, ranging from 40 to 100%. The distance from hibernacula to the water’s edge ranged from locations immediately adjacent to water to a maximum of 12.7 m. Soil moisture exhibited a negative relationship with distance from the waterfront, indicating that soil became progressively drier with increasing distance from water (Table 2, Figure 6).

### 3.2. Hibernation and Emergence from Hibernation

In the field, the last observation of an individual entering hibernation occurred on 29 November, when temperature was 3.2~14.7 (Mean 10.2 ± 3.6) °C, and the first observation of a frog emerging from hibernation was recorded on 29 March of the following year, when temperature was 0.1~17.1 (Mean 8.7 ± 6.4) °C (Table 3). Thus, the field hibernation period lasted 121 days.

**Figure 7:**
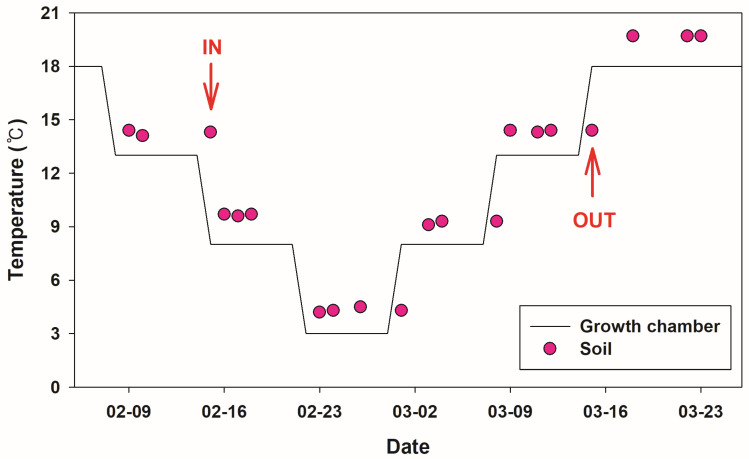
The timing of hibernation onset and release according to temperature changes in artificial hibernation experiments. Solid line means growth chamber temperature and red solid indicates soil temperature. Arrows labeled “IN” and “OUT” indicate the timing of entry into and emergence from hibernation, respectively.

Laboratory experiments yielded results that differed somewhat from those observed in the field. In the artificial hibernation experiment, all frogs initiated burrowing and entered hibernation at a soil temperature of 14.3 °C. During the subsequent warming phase, locomotor activity resumed when the temperature reached 14.4 °C (Figure 7). Mortality was observed in both field and laboratory settings. During the 2022–2023 field season, 5 of 31 hibernating individuals died, resulting in a mortality rate of 16.1%. In the laboratory experiment, 2 of 35 frogs died, corresponding to a mortality rate of 5.7%. In the Aquatic Botanical Garden, most deceased frogs were found in areas adjacent to the water (Figure 1).

## 4. Discussion

Hibernation plays a critical role in the life history of most amphibians. It influences growth, body condition, maturation and reproduction [17,18]. Because hibernation involves physiological changes and hormonal regulation, it is closely linked to breeding. For example, in *Rana muscosa*, only individuals that had undergone hibernation were capable of reproduction, whereas those that did not hibernate failed to breed [19]. In captive-bred populations, individuals that experience hibernation showed higher post-release survival rates in the wild [20]. Thus, hibernation is not only essential for maintaining individual health and survival but also crucial for long-term population persistence through successful reproduction.

In this study, we found that subadults and larger individuals of *P. chosenicus* participated in terrestrial hibernation. Considering previously reported growth patterns of the species [21,22], frogs generally reach a SVL of approximately 30.0 mm by October. In the congeneric *P. caralitanus*, juveniles can similarly attain a body size exceeding 30.0 mm within their first year [23]. These body sizes correspond to subadult stages capable of engaging in terrestrial hibernation. Our findings therefore suggest that all individuals involved in reproduction, including subadults, participate in hibernation, and that the restored population in the Aquatic Botanical Garden has acquired an ecological structure that facilitates stable population maintenance.

*P. chosenicus* primarily entered hibernation between late October and late November as air temperature declined, consistent with previous reports that hibernation begins in late autumn [24]. The number of hibernacula increased from November to December and remained relatively stable until March, when rising soil temperatures rose together with air temperature, after which the number of hibernating individuals declined as frogs emerged. From November to January, soil temperatures were consistently higher than air temperatures, suggesting that *P. chosenicus* takes advantage of the thermal buffering capacity of soil to mitigate exposure to extreme winter cold.

Two individuals were observed moving between hibernacula during the hibernation period (Figure 1). These movements are likely to have occurred when environmental conditions at the original hibernaculum became unsuitable or when external changes posed a threat to survival, prompting frogs to accept the associated risk and relocate. Holenweg and Reyer [1] reported that two Rana species moved particularly when temperatures increased after hibernation, and Doi et al. [25] also documented similar movements. These behaviours were interpreted as responses to suboptimal temperatures for survival, despite the associated energetic costs. Likewise, our results suggest that *P. chosenicus* may relocate hibernation sites in response to changes in temperature or other environmental conditions. Such movements likely aim to secure more favourable microhabitats, indicating that this species exhibits adaptive behaviour in selecting and switching hibernation sites and employs flexible strategies to enhance survival under fluctuating environmental conditions.

We implanted PIT tags in a total of 408 individuals; however, this study confirmed terrestrial hibernation sites for only 49 individuals. The study site is an open ecosystem, and the presence of a channel flowing immediately to the west, which is connected to the study area, suggests a high potential for individuals to disperse or leave the site. Furthermore, according to AmphibiaWeb, the genus *Pelophylax* is generally known to hibernate in water, with only some individuals utilizing terrestrial sites. Therefore, it is highly likely that some individuals not tracked in this study overwintered in aquatic habitats.

In our study, *P. chosenicus* burrowed to depths of up to 23 cm, where soil temperature was significantly higher than air temperature. This indicates that burial depth plays an important protective role against temperature fluctuations during hibernation. Shallow burials leave frogs more exposed to freezing and increase the risk of mortality due to freezing, whereas deeper burials provide greater thermal stability in winter. Some terrestrial toads are known to burrow deeper than 1 m [3,4]. In Rana species, hibernation has been reported at depths of around 3–7 cm [1]. In the congeneric Tokyo daruma pond frog *P. porosus porosus*, PIT telemetry studies have shown that frogs burrow to an average depth of 19.8 cm, similar to our findings. In both species, hibernation depth varies with temperature, and the same pattern of depth adjustment was observed in *P. porosus porosus* [26]. Therefore, the ability of *P. chosenicus* to adjust burial depth during hibernation is therefore likely one of its key strategies for surviving extreme environmental conditions. The observed tendency for increased hibernation depth under lower winter temperatures highlights the species’ adaptation to cold stress during hibernation.

*P. chosenicus* generally has limited daily movement, typically within 10 m, and spends its life near breeding sites, with relatively small home ranges [24,27]. Consistent with this, most terrestrial hibernation (excluding aquatic hibernation) in this study occurred near the Aquatic Botanical Garden. Of the 52 hibernacula identified, 43 (82.69%) were located within 10 m of the water’s edge. Although this distribution may partly reflect limited movement capacity, soil moisture during hibernation appears to be an important factor in hibernation site selection. Many ranid frogs hibernate underwater to avoid desiccation stress [2]. *P. chosenicus* uses both aquatic and terrestrial hibernation, and when selecting terrestrial hibernacula, frogs mainly chose locations with soil moisture above 70%, likely to prevent dehydration during winter. Consequently, *P. chosenicus* appears to prefer hibernacula close to the water’s edge, where soil moisture is higher. Because amphibians absorb water through the skin during hibernation, hibernating in dry environments can exacerbate water loss and negatively affect survival. The preference of *P. chosenicus* for high-moisture soil can therefore be interpreted as a strategy to avoid dehydration during hibernation.

Temperature is a major determinant of hibernation duration [28]. In both field and laboratory settings, frogs initiated hibernation when soil temperature exceeded air temperature, and terminated hibernation when air temperature exceeded soil temperature. As ectothermic animals, frogs are inherently sensitive to thermal changes [2]. Future studies aimed at conserving *P. chosenicus* should therefore include investigations of physiological mechanisms underlying responses to thermal regimes. In Rana species, overwinter survival has been shown to be closely associated with winter severity, with cold spells and rapid temperature fluctuations increasing mortality [29]. Longer and colder hibernation periods also reduce survival [30]. In our study, mortality of *P. chosenicus* occurred in both field and laboratory settings, and these deaths were likely influenced by temperature variation and hibernation duration.

## 5. Conclusions

This study demonstrates that environmental factors play a critical role in the selection of hibernation sites by *P. chosenicus*. In particular, hibernacula that satisfy specific conditions—sufficient soil depth, high soil moisture and proximity to water—are key to enhancing individual survival and, consequently, population persistence. These findings can therefore serve as valuable reference data for habitat restoration efforts targeting this species. When designing microhabitats for hibernation, restoration plans should include measures to (1) secure soils of suitable depth in areas close to the water’s edge and (2) maintain high-moisture soil conditions.

Ongoing climate change is likely to alter the survival prospects of *P. chosenicus*. Warmer winters may reduce energy expenditure and improve body condition, potentially increasing the probability of reproductive success in the subsequent breeding season [31,32,33]. However, rising temperatures alone cannot be assumed to simply increase population size, because climate change also affects many other factors, including the prevalence of viruses and diseases. To ensure the long-term persistence of *P. chosenicus*, it will be necessary not only to protect its hibernation sites but also to develop flexible, adaptive strategies that account for broader climatic impacts.

## Figures and Tables

**Table 1:** Average environmental variables of hibernation sites by period (1st: November 2021–March 2022, 2nd: November 2022–March 2023, 3rd: November 2023–March 2024).

Variable	1st (n: 6)					2nd (n: 61)					3rd (n: 47)				
	Nov (n: 1)	Dec (n: 1)	Jan (n: 1)	Feb (n: 2)	Mar (n: 1)	Nov (n: 0)	Dec (n: 16)	Jan (n: 16)	Feb (n: 15)	Mar (n: 14)	Nov (n: 7)	Dec (n: 14)	Jan (n: 9)	Feb (n: 9)	Mar (n: 8)
SVL (mm)	38.7	55.5	58.0	46.7 ± 7.6	53.1	-	43.7 ± 8.6	46.4 ± 7.8	46.6 ± 8.8	49.2 ± 8.3	46.4 ± 7.2	43.8 ± 8.3	44.2 ± 7.1	42.4 ± 2.8	42.9 ± 3.7
	49.8 ± 7.8 (38.7~58.0)					46.4 ± 8.6 (32.5~64.9)					43.8 ± 6.6 (29.3~61.8)				
BW (g)	7.7	22.4	23.4	14.8 ± 7.4	21.8	-	13.8 ± 9.5	15.1 ± 9.2	15.6 ± 9.6	18.1 ± 10.5	14.2 ± 7.2	12.7 ± 8.3	13.2 ± 7.6	11.1 ± 2.6	10.1 ± 2.0
	17.5 ± 7.0 (7.4~23.4)					15.6 ± 9.8 (5.2~40.0)					12.3 ± 6.6 (3.5~33.1)				
Soil depth (cm)	5.0	6.0	18.0	11.0 ± 5.0	11.0	-	9.9 ± 4.4	11.7 ± 5.3	12.5 ± 4.9	8.8 ± 6.9	10.3 ± 3.7	11.6 ± 5.3	15.8 ± 4.5	9.1 ± 3.7	10.2 ± 3.1
	10.3 ± 5.1 (5.0~18.0)					10.7 ± 5.6 (1.0~23.0)					11.5 ± 4.9 (3.5~21.0)				
Soil temperature (°C)	-	-	-	-	-	-	5.9 ± 1.1	2.1 ± 1.7	4.9 ± 0.8	10.1 ± 1.7	7.9 ± 0.7	10.8 ± 0.4	3.5 ± 1.7	5.3 ± 0.3	9.5 ± 0.8
						5.6 ± 3.2 (0.2~13.8)					7.7 ± 3.0 (1.2~11.6)				
Soil pH	-	-	-	-	-	-	6.0 ± 0.3	6.3 ± 0.2	6.2 ± 0.2	6.2 ± 0.4	6.0 ± 0.2	6.4 ± 0.2	6.6 ± 0.3	6.5 ± 0.1	6.4 ± 0.3
						6.2 ± 0.3 (5.5~6.8)					6.4 ± 0.3 (5.8~7.0)				
Soil moisture (%)	-	-	-	-	-	-	87.4 ± 11.6	78.8 ± 13.0	78.3 ± 17.4	81.3 ± 16.3	82.1 ± 13.1	74.9 ± 9.1	70.0 ± 11.3	71.3 ± 5.8	71.4 ± 18.3
						81.5 ± 15.1 (40.0~100.0)					73.7 ± 12.4 (40.0~100.0)				
Distance from the waterfront(m)	1.0	6.0	6.0	5.5 ± 0.5	6.0	-	1.8 ± 2.9	2.9 ± 4.0	5.0 ± 4.5	2.7 ± 3.0	1.6 ± 1.5	5.6 ± 4.9	5.9 ± 4.7	5.6 ± 4.8	6.1 ± 4.6
	4.0 ± 2.2 (1.0~6.0)					3.4 ± 4.0 (0~12.7)					5.1 ± 4.8 (0~11.6)				
Avg daily temperature (°C)	8.3	7.4	−3.6	−1.0	5.9	-	3.1	2.8	2.3	9.4	6.3	8.5	−0.9	3.4	5.8
	2.7 ± 4.7 (−3.6~8.3)					4.3 ± 2.9 (2.0~9.4)					4.9 ± 3.3 (−0.9~8.5)				

**Table 2:** Results of correlation analysis between each variable (Correlation coefficient).

Variable	BW (g)	Soil Depth (cm)	Soil Temp (°C)	Soil pH	Soil Moisture (%)	Distance from the Waterfront (cm)	Avg Daily Temp (°C)
SVL (mm)	1.0 **	−0.1	0.0	0.0	0.0	0.1	0.1
BW (g)		−0.1	0.0	0.0	0.0	0.1	0.1
Soil depth (cm)			−0.3 **	0.1	−0.1	0.1	−0.2 *
Soil temperature (°C)				0.0	0.1	0.0	0.8 **
Soil pH					−0.5 **	0.3 **	−0.1
Soil moisture (%)						−0.3 **	0.1
Distance from the waterfront (cm)							0.0

* *p*-Value < 0.05, ** *p*-Value < 0.01.

**Table 3:** Comparison of artificial hibernation in the laboratory and natural hibernation in the field.

Variable	Field	Laboratory
Hibernation start and release dates (Duration)	29 November 2022–29 March 2023 (121 days)	3 February 2022–25 March 2022 (50 days)
Last individual hibernation start temperature (°C)	3.2~14.7 (Mean 10.2 ± 3.6)	14.3
First individual hibernation release temperature (°C)	0.1~17.1 (Mean 8.7 ± 6.4)	14.4
Number of Dead Individuals (Mortality Rate)	5 dead out of 31 individuals (16.1%)	2 dead out of 35 individuals (5.7%)

## Data Availability

The raw data supporting the conclusions of this article will be made available by the authors on request.
